# Negative Effects of a Multimodal Inpatient CBASP Program: Rate of Occurrence and Their Impact on Treatment Outcome in Chronic and Treatment-Resistant Depression

**DOI:** 10.3389/fpsyt.2021.575837

**Published:** 2021-08-09

**Authors:** Philipp Herzog, Sophia Häusler, Claus Normann, Eva-Lotta Brakemeier

**Affiliations:** ^1^Department of Clinical Psychology and Psychotherapy, Philipps-Universität Marburg, Marburg, Germany; ^2^Department of Psychiatry and Psychotherapy, Universitätsklinikum Freiburg, Freiburg, Germany; ^3^Center for Mind, Brain and Behavior, University of Marburg and Justus Liebig University Giessen, Marburg, Germany; ^4^Psychosomatic Clinic, Schön Klinik Bad Arolsen, Bad Arolsen, Germany

**Keywords:** negative effects, inpatient psychotherapy, chronic depression, cognitive behavioral analysis system of psychotherapy, treatment outcome, dependence, CBASP

## Abstract

**Background:** A growing number of studies indicate that the Cognitive Behavioral Analysis System of Psychotherapy (CBASP) is effective in treating chronic depression. However, there is no systematic research into possible negative effects. Therefore, the objectives of the study were to investigate the rate of occurrence of negative effects of an inpatient CBASP program and their impact on treatment response.

**Methods:** Patients with chronic depression and treatment resistance who completed the 12-week multimodal inpatient CBASP treatment program in an open trial (*N* = 52) retrospectively completed the Inventory for the Assessment of Negative Effects of Psychotherapy (INEP) during follow-up data collection. Severity of depressive symptoms was assessed self- and observer-rated at admission, discharge, and 6 months follow-up. Rates of occurrence of negative effects were calculated and binary logistic regression analyses were conducted to determine the relationship to treatment outcome.

**Results:** The results indicate that 92.3% of patients reported having experienced at least one negative effect and 45.2% indicated dependence on their therapist. Stigmatization and financial concerns as well as intrapersonal changes were reported by about one-third. Only dependence on the therapist negatively impacted treatment outcome in both outcome measures.

**Conclusions:** While almost all patients reported at least one negative effect of a multimodal inpatient CBASP treatment program, most of the reported negative effects appear to be benign. However, dependence on the therapist seems to have a negative impact on treatment outcome. If these results can be replicated in future large-scale, randomized controlled prospective studies, CBASP therapists should be aware of possible dependence and consciously address it during treatment.

## Introduction

Systematic research to assess and report negative effects such as side effects and other unwanted effects of psychotherapy is lacking (1). By definition, negative effects of psychotherapy can be divided into unwanted effects caused by malpractice or unethical behavior and unwanted effects caused by correct treatment (referred to as side or adverse effects) (2). The relevance of negative effects depends on the severity and duration and should therefore be considered in relation to the short- and long-term treatment outcome: Negative effects are considered relevant, in case they negatively relate to treatment outcome; and irrelevant, if there is no or a positive association to treatment outcome (2).

Over the past 10 years, several instruments for assessing negative effects of psychotherapy have been developed and partially validated, notably the Inventory for the Assessment of Negative Effects of Psychotherapy [INEP; (3)] and the Negative Effect Questionnaire [NEQ; (4)]. According to the INEP, a recent study reveals that 58.7% of patients from a psychiatric hospital and 45.2% of patients from a psychosomatic hospital reported to have experienced at least one negative effect during therapy (5). Another INEP study indicates that 93.8% of former psychotherapy patients reported having experienced at least one negative effect during or after psychotherapy, with the highest rates concerning intrapersonal changes, stigmatization, and relationships (3). In a recent inpatient study, which did not use INEP to measure negative effects, 60–65% of psychiatric inpatients reported deterioration of mood state and unwanted treatment reactions; unwanted treatment reactions decreased in the course of treatment but were negatively associated with the treatment outcome (6). In addition, first research data indicate that negative effects have a negative impact on the outcome of treatment for obsessive–compulsive disorder (7). Overall, knowledge about the occurrence of specific negative effects in different treatment settings and their effects on treatment outcomes is too limited to determine the relevance of the negative effects. However, these reported high rates of occurrence of negative effects of psychotherapy in different treatment settings and mental disorders underline the importance of further investigations of negative effects, especially in seriously burdened patients like those suffering from treatment-resistant chronic depression (CD).

The Cognitive Behavioral Analysis System of Psychotherapy [CBASP; (8, 9)] is a disorder-specific treatment for patients with CD. Since chronically depressed patients have often experienced childhood maltreatment (10), the main goal of therapy is to enable patients to experience healing relationships. Through disciplined personal involvement, the therapist discloses her/his positive and negative personal feelings and reactions that the patient triggers in her/him, to teach the patient that people today respond to him differently than she/he expected or feared, supported by interpersonal discrimination exercises (11). CBASP can therefore be described as an interpersonal learning therapy. Several research studies indicate the efficacy of CBASP as an outpatient treatment for CD (12, 13) and CBASP as an inpatient treatment program (14).

In general, a strong therapeutic alliance has consistently been associated with positive treatment outcomes: Meta-analyses revealed a positive alliance–outcome association for face-to-face and internet-based psychotherapy with a medium and significant effect, explaining about 8% of the variability in treatment outcome (15, 16). More specifically, a relationship between the therapeutic alliance and outcome in CBASP has also been well-documented in literature (17, 18). Indeed, an early positive therapeutic alliance predicted favorable outcomes in CBASP (19) and independently contributed to specific CBASP elements to depressive symptom improvements, yielding unique and additive effects to the outcome (20). In line, larger depressive symptom improvement was related to a higher emphasis on the therapeutic relationship during CBASP (21). Mechanistically, Constantino et al. (22) showed that higher therapeutic alliance predicted decreases in hostile–submissive behavior, which, in turn, predicted less depressive symptoms in patients treated with CBASP. In line with this, decreases in patients' hostile–submissive behavior were significantly associated with a reduction of depressive symptoms and favorable treatment response (23).

However, in addition to these positive effects, it appears important to investigate negative effects of CBASP as well. Preliminary results of a self-constructed, non-validated questionnaire to assess side effects of a multimodal inpatient CBASP treatment program provided some interesting findings; however, the interpretation and generalizability of these results are hampered by methodological limitations of the questionnaire (24). Thus, research data with validated questionnaires (such as INEP) for specific negative effects during CBASP in CD and their relation to treatment response are lacking. In addition, it is of high clinical interest to further investigate negative effects in inpatient treatment, as inpatient treatment might trigger specific negative effects due to its short but intensive treatment (6).

Therefore, the objectives of the current study were to exploratively investigate (1) the rates of occurrence of negative effects of a multimodal inpatient CBASP treatment program and (2) the impact of specific negative effects on the clinician- and self-rated treatment response in order to determine their relevance.

## Materials and Methods

The study was conducted at the Affective Research Unit of the Department of Psychiatry and Psychotherapy, University of Freiburg Medical School, and approved by the Ethics Committee of the University of Freiburg. It has been performed in accordance with the ethical standards laid down in the 1964 Declaration of Helsinki and its later amendments. All patients gave their written informed consent prior to their inclusion in the study. However, this pilot study has unfortunately not been pre-registered. The present study is part of a larger research project of which the feasibility and outcome data have already been published (14, 25).

### Patients

Seventy consecutive patients who met the inclusion criteria of suffering from CD according to DSM-IV, aged 18–70 years, fluently speaking German, and being resistant to outpatient treatment were enrolled in the CBASP inpatient program. Treatment resistance was defined as fulfilling the criterion for either medication resistance (no response to two or more adequate trials of antidepressants) according to Thase and Rush (26) and/or psychotherapy resistance (no response to at least two health-insurance-reimbursed psychotherapies with at least 22 sessions each). Exclusion criteria were defined as a history of bipolar I disorder, comorbid substance dependency with <3 months of abstinence, antisocial personality disorder, severe forms of autism, and mental disorders due to organic factors according to the DSM-IV criteria. Of the 70 patients, 65 completed the study [dropout rate 7.1%; reasons for dropout: serious conflicts with other patients (three patients), severe psychosocial problems impossible to handle due to the distance to the patient's hometown (one patient), and diagnosis of mild cognitive impairment after 3 weeks of treatment (one patient)] [cf. (14)]. Completers (*n* = 52, 80% retention) filled out the INEP (3) between 6 and 12 months after discharge. No significant differences (*p* > 0.05) were found between completers who filled out the INEP and completers who did not (*n* = 13, 20%). The dropout patients refused to fill out follow-up questionnaires. The temporal variance of 6 to 12 months is due to the fact that this research question was developed after the study had already been designed (see **Limitations and Future Research**). In this manuscript, we analyze the data of the 52 patients who completed INEP.

### Study Treatment: Multimodal Inpatient CBASP Treatment Program

The 12-week multimodal inpatient CBASP treatment program portrayed in this study is based on the CBASP treatment by McCullough (8) and was established in 2008. More specifically, this CBASP treatment program has been modified and manualized for inpatient use (27) and now includes the following CBASP-specific treatment elements [cf. (14)]: individual psychotherapy in CBASP sessions (two 50-min sessions per week; use of all CBASP strategies), CBASP group psychotherapy (two 90-min sessions per week, particularly focusing on the application of situational analyses including Kiesler Circle), CBASP body and movement therapy (one 60-min session per week, body-related exercises through various Kiesler Circle Training exercises), CBASP nursing staff sessions (one 30-min session per week; repetition of core treatment elements as well as exercises and role-plays to refresh the content of the CBASP individual sessions and group therapies), occupational group therapy (two 90-min sessions per week; art-related treatment of CBASP-relevant topics, e.g., significant others), and social counseling sessions (as needed, but at least one 30-min session per week; support in managing/resolving interpersonal and psychosocial problems such as divorce or job changes). As indicated, patients received two sessions per week over the course of 12 weeks leading to a total session number of 24 sessions on average exceeding the minimum of 18 sessions at least for CD (28). Of note, as a modification to the outpatient treatment, transference hypotheses are formulated not only for the individual therapist but also for the treatment ward team and for the patient group. In addition to this intensive inpatient treatment program, patients were able to participate in non-CBASP-specific sports and occupational therapies. Moreover, all patients received algorithm-based pharmacotherapy in accordance with current national and international guidelines for the treatment of depression (29, 30) and according to clinical experts supervision.

As discharge and the time thereafter generally play a major role in the success of the inpatient setting, the last 2 weeks of the multimodal inpatient CBASP treatment program focused on relapse prevention and follow-up by making arrangements for discharge from the hospital and continuation of treatment in the outpatient setting (in the form of a discharge plan). If patients continued to use the CBASP strategies they had learned and wanted a further treatment option in the multimodal inpatient CBASP treatment program, they could attend a 4-week inpatient CBASP refresher course at least 6 months after their first discharge. In addition, in at least some cities, CBASP support groups for patients were established to prevent relapse after discharge (27).

Among all patients, about 80% of the patients underwent outpatient psychotherapy after discharge. Of those, the percentual distribution of the therapy orientation is as follows: 46% cognitive behavioral therapy, 32% CBASP, 8% psychodynamic therapies, and 2% client-centered psychotherapy. In 18% of the patients, the continuation of a psychotherapy already started overlapping within the inpatient treatment; in 46% of the patients, the psychotherapy was still running at the time of the follow-up interview. Moreover, 42% of the patients visited the CBASP self-help groups established in Freiburg.

### Measures

#### Inventory for the Assessment of Negative Effects of Psychotherapy

INEP is a self-report questionnaire assessing the negative effects of psychotherapy. Precisely, INEP records experiences and changes that patients have experienced in themselves and in their interaction with other people after the completion of their psychotherapy (3). The 21-item scale covers seven domains where negative effects may occur: “intrapersonal changes,”^1^ “dependence,” “family,” “friends,” “partnership,” “stigmatization and financial concerns,” and “malpractice.” For example, the key items that measure dependence are formulated as follows: “During therapy and/or after its completion, it is harder for me to make important decisions on my own” and “During therapy and/or after its completion, I feel addicted to my therapist.” The key items measuring intrapersonal changes are phrased like this: “Since the end of my therapy, I suffer less/more from the events of my past compared to the time before the therapy,” “During therapy and/or after its completion, I've had long periods of bad times,” and “During therapy and/or after its completion, I had suicidal thoughts/intentions for the first time.” Concerning malpractice, key items are as follows: “I felt hurt by the therapist's statements,” “My therapist forced me to do things (exposure, role plays, etc.) that I didn't really want to do,” or “During the therapy there were direct sexual assaults by my therapist.” Patients were asked to indicate their agreement or disagreement with these statements on a four- or three-point Likert scale. In addition, patients must indicate for each item whether they attribute this change to psychotherapy or other life circumstances.

INEP in its final version has demonstrated good internal consistency (α = 0.86), while the original subscale “malpractice” showed only satisfactory internal consistency (α = 0.73). Initial results of factor analysis showed a seven-factor solution that supports its construct validity (3). In our sample, the total scale showed equally good internal consistency as indicated by Cronbach's alpha of α = 0.86, while the subscale “negative effects” yielded a Cronbach's alpha of α = 0.84, and, respectively, the subscale “malpractice,” α = 0.78 (excluding items 17, 18, and 19 due to no variance in our sample), both indicating good internal consistency. During follow-up data collection, INEP was assessed between 6 and 12 months after discharge.

#### 24-Item Version of the Hamilton Rating Depression Scale

HRSD-24 is the 24-item version of the well-established clinician-rated Hamilton Rating Scale for Depression assessing the symptom severity of depression and served as primary outcome measure (31). Each item is rated from 0 to 2 or 0 to 4, total score is reported as a sum score and ranges from 0 to 76, while higher sum scores indicate more severe depressive symptoms. HRSD-24 was assessed at intake, discharge, and at 6 months follow-up by blinded and trained raters. *A priori*, treatment response was defined as a decrease in symptom severity of at least 50% in the HRSD-24. While the HRSD-24 showed only an acceptable internal consistency as indicated by Cronbach's alpha of α = 0.63 in our sample, this instrument showed in general a good internal consistency indicated by a Cronbach's alpha of α = 0.79 in other studies (32).

#### Beck Depression Inventory-II

BDI-II is an internationally widely used 21-item self-report questionnaire measuring somatic, cognitive, and affective symptoms of depression (33). It serves as a secondary outcome measure in the present study. Scores are ranging from 0 to 63, with higher values indicating more severe depressive symptoms. Like the HRSD-24, BDI-II was assessed at intake, discharge, and at 6-month follow-up. Treatment response was *a priori* defined in the same way as for HRSD-24, that is, a decrease in symptom severity of at least 50% of the BDI-II sum score. In line with internal consistency estimations reported in literature (34), the BDI-II yielded a Cronbach's alpha of α = 0.88, indicating a very good internal consistency in our sample.

#### Other Baseline Measures

At the beginning of the study (baseline), sociodemographic questions were asked by a self-report questionnaire including age, gender, educational level, and marital status. Clinical characteristics were also assessed, including diagnosis of CD according to DSM-IV, early onset of depression, age at onset, inpatient treatment and psychotherapy in the past, medication and psychotherapy resistance, and suicide attempts in the past. Finally, axis I and axis II comorbidities were assessed with SCID I (35) and SCID II (36).

### Statistics/Statistical Analyses

A data screening according to the suggestions of Tabachnick and Fidell (37) and a test of the assumptions of logistic regression were carried out (37). The data screening showed that between 6.2% and 36.9% of the variables used to measure treatment outcome were missing. In BDI-II, 6.2% of the data were missing at baseline. After completion of the treatment, 7.7% of the data were missing, and 6 months later, 35.4% of the data were missing. Regarding HRSD-24, all data were available for measurements at baseline and discharge. Six months after discharge, 6.2% of the HRSD-24 data were missing. The Little MCAR test (38) was performed to analyze missing values. The results were not significant, implicating that missing values appeared random. According to Tabachnick and Fidell (37), missing values were estimated using the expectation maximization (EM) procedure. Correlational analyses between demographic variables (i.e., gender, age, education, and relational status) and negative effects as indicated by the factors of the INEP were computed using Spearman-Rho correlations. To investigate the relationship between negative effects of psychotherapy and individual treatment response, a binary logistic regression was calculated using the backward stepwise method (the Backward:LR method). Two important assumptions for logistic regression (linearity in logistic regression and the absence of multicollinearity) were fulfilled. In order to evaluate the contribution of a single predictor to the model, the Wald test was calculated. The efficiency coefficient Exp(*B*), also called odds ratio (OR), and its confidence intervals were calculated to evaluate the effect of the predictor variables. All analyses were conducted using SPSS, version 21 (39). *z*-test *post-hoc* power analyses (two-tailed) were calculated using G^*^Power 3.1 (40, 41).

## Results

### Patient Characteristics

The mean age at baseline of the 52 patients was 48.1 years (SD = 10.1 years); 61.5% were female. The mean patient sum score of the HRSD-24 at baseline was 31.3 (SD = 6.4), while the mean BDI-II sum score at baseline was 33.6 (SD = 10.5), both indicating severe depression. Moreover, the criteria for medication and psychotherapy resistance were each fulfilled by 88.5% of the sample. Further relevant sociodemographic and clinical characteristics are depicted in Table 1.

**Table 1:** Sociodemographic characteristics of the sample (*N* = 52).

**Characteristics**	**Patients (*N* = 52)**
Age at entry, *M* (SD)	48.1 (10.1)
**Sex**, ***n*****(%)**	
Male	20 (38.5)
Female	32 (61.5)
**Educational level**, ***n*****(%)**	
No educational degree	4 (7.7)
Primary education	27 (51.9)
Secondary education	3 (5.8)
Higher education	18 (34.6)
**Marital status**, ***n*****(%)**	
Single	11 (21.2)
Married/couples relationship	29 (55.8)
Divorced/in separation	12 (23.1)
**Diagnosis of Chronic Depression (DSM-IV^a^), *n* (%)**	
Double Depression	24 (46.2)
Recurrent Major Depression	17 (32.7)
Chronic Major Depression	11 (21.2)
Early onset of depression^b^, *n* (%)	42 (80.8)
Age at onset *M* (SD)	15.0 (10.5)
Comorbid Axis I disorder^c^, *n* (%)	43 (61.4)
Comorbid Axis II disorder^d^, *n* (%)	47 (67.1)
Inpatient treatment in the past^e^, *n* (%)	43 (82.7)
Psychotherapy in the past^f^, *n* (%)	50 (96.2)
Medication resistance^g^, *n* (%)	46 (88.5)
Psychotherapy resistance^h^, *n* (%)	46 (88.5)
Suicide attempt in the past, *n* (%)	19 (36.5)
HRSD-24 score at baseline, *M* (SD)^i^	31.3 (6.4)
BDI-II score at baseline, *M* (SD)^j^	33.6 (10.5)

*M, mean; SD, standard deviation; n, number*.

a *DSM-IV, Diagnostic and Statistical Manual of Mental Disorders, 4th Edition.*

b *Before the age of 21.*

c *Assessed with Structured Clinical Interview (SCID) I (22).*

d *Assessed with Structured Clinical Interview (SCID) II (23).*

e *Inpatient treatment in a psychiatric or psychosomatic hospital.*

f *Treatments with minimum 22 sessions.*

g *No response to two or more adequate trials of antidepressants.*

h *No response to two or more health insurance-reimbursed psychotherapies with each minimum 22 sessions in Germany.*

i *HRSD-24, Hamilton Rating Depression Scale, 24 Items, scale 0–75 (20).*

j *BDI-II, Beck Depression Inventory-II, 21 Items, scale 0–63 (21)*.

### Rates of Occurrence of Reported Negative Effects

**Figure 1:**
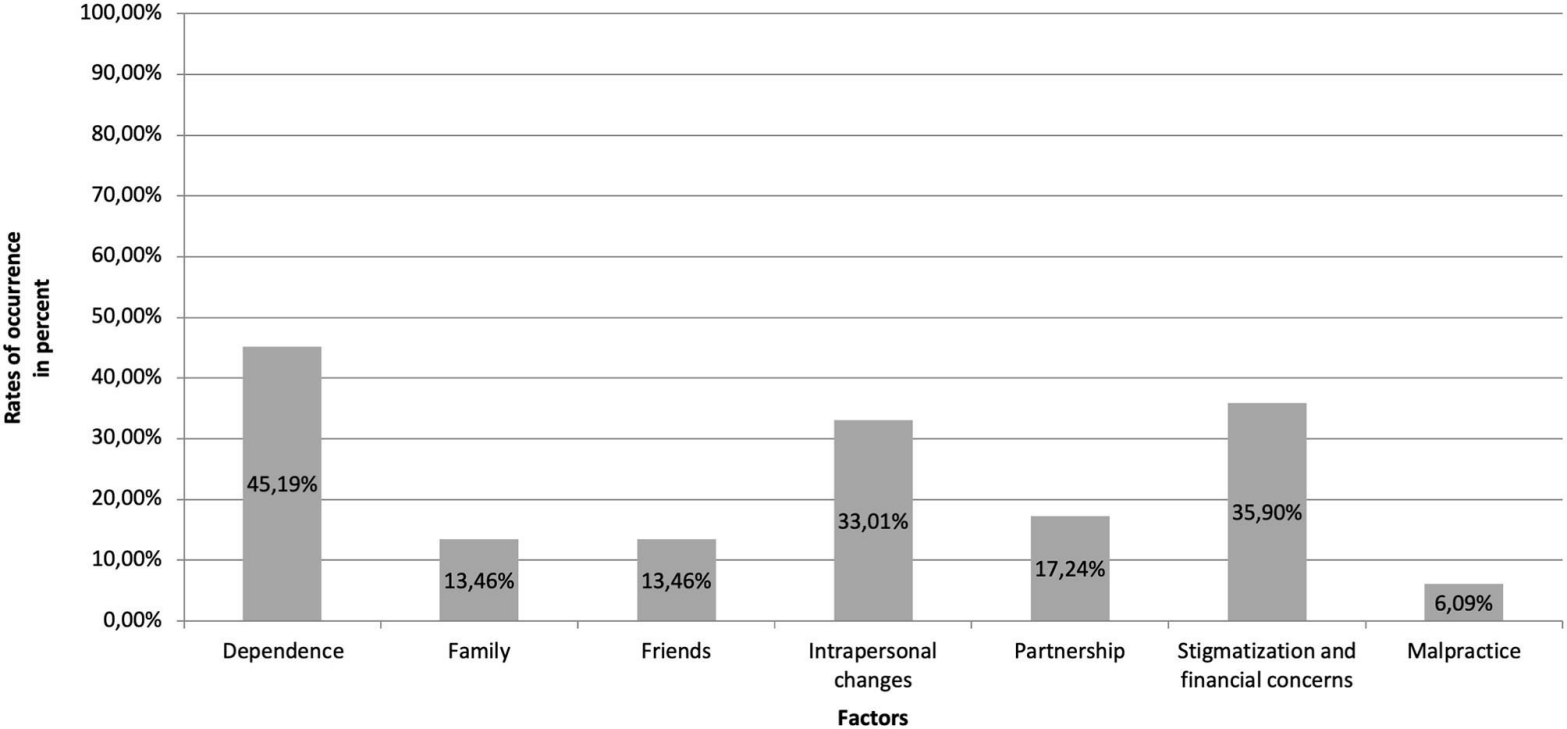
Rates of occurrences of reported negative effects of psychotherapy according to the seven factors of the INEP (3).

The 21-item scale covers seven domains of negative effects: “intrapersonal changes” (*M* = −0.22, SD =.59, Min = −1.33, Max = 1.33), “dependence” (*M* = 0.21, SD = 0.39, Min = 0, Max = 1.5), “family” (*M* = −0.72, SD = 1.11, Min = −3.0, Max = 1.0), “friends” (*M* = −0.55, SD = 1.00, Min = −3.0, Max = 1.0), “partnership” (*M* = −0.10, SD = 0.52, Min = −1.5, Max = 1.5), “stigmatization and financial concerns” (*M* = 0.09, SD = 0.34, Min = 0, Max = 2.33), and “malpractice” (*M* = 0.08, SD = 0.21, Min = 0, Max = 1.33). Figure 1 presents the rates of occurrences of the seven INEP domains of negative effects caused by therapy. According to INEP, 92.3% reported having experienced at least one negative effect. Regarding the different domains, 45.2% reported having experienced dependence on their therapist. Experiences of stigmatization and financial concerns were reported by 35.9%, while intrapersonal changes in terms of symptom deterioration were experienced by 33.0% of patients. Furthermore, the lowest rates of negative effects were reported concerning family (13.5%), friends (13.5%), and partnership (17.2%). Some patients (6.1%) reported malpractice, with this comparatively high figure resulting from items stating that their therapist forced them to do things they did not want to do (such as role-playing) (two patients partly agreed, five patients agreed somewhat) and that patients felt hurt by therapists' statements (one patient totally agreed, one partly agreed, and eight agreed somewhat). No patient reported sexual abuse, physical assault, or other misconduct.

The Spearman-Rho correlation indicates that the factor “dependence” correlates non-significantly with sex (ρ = −0.20, *p* > 0.05), age (ρ = −0.16, *p* > 0.05), and educational status (ρ = −0.14, *p* >0.05), and significantly with marital status (ρ = −0.35, *p* < 0.05), indicating a small to medium effect. Apart from these findings, only the factor “family” shows significant correlations with age (ρ = −0.33, *p* < 0.05) and educational status (ρ = 0.34, *p* < 0.05). The factors “intrapersonal changes,” “friends,” “partnership,” “stigmatization and financial concerns,” and “malpractice” did not show any significant correlational relationship with these demographics (all *p* > 0.05).

### Prediction of Treatment Response Measured by HRSD-24

According to the HRSD-24 criterion for treatment response, 46 of the 52 patients (88.5%) responded to the 12-week multimodal inpatient CBASP treatment program. Six months after discharge, 32 patients (61.5%) still reached the response criterion. The results of the last step of the binary logistic regression using the backward stepwise method for treatment response regarding HRSD-24 are depicted in Table 2. The factor “intrapersonal changes” appears negatively related to treatment response at posttreatment [*b* = −0.36, Wald $\chi_{\left(1\right)}^{2}$ = 5.05, *p* = 0.03]. The Exp(*B*) value indicates that when “intrapersonal changes” are increased by one unit, the odds ratio is.70 times as large and therefore patients are 30% less likely to respond to treatment. However, after 6-months of follow-up, the factor “dependence” is significantly associated with treatment response (*b* = −1.02, Wald $\chi_{\left(1\right)}^{2}$ = 5.08, *p* = 0.02). The Exp(*B*) value indicates that when “dependence” is increased by one unit, the odds ratio is 0.36 times as large and therefore patients are 64% less likely to respond to treatment. Other factors were not significantly associated with treatment response (*p* > 0.05).

**Table 2:** Results of the logistic regression of negative effects on treatment response at discharge (T2) and follow-up 6 months after discharge (T3) measured by Hamilton Rating Depression Scale, 24-item version (HRSD-24).

**Variables**	***B***	**Wald**	***p***	**Exp(*B*)**	**95.0% CI for Exp(** ***B*** **)**	
					**Lower**	**Upper**
**Criterion: Response on** ***HRSD-24*** **T2**						
Family	−1.90	1.93	0.17	0.15	0.01	2.19
Intrapersonal changes	−0.36	5.05	0.03^*^	0.70	0.51	0.96
**Criterion: Response on** ***HRSD-24*** **T3**						
Dependence	−1.02	5.08	0.02^*^	0.36	0.15	0.88
Malpractice	0.56	1.61	0.20	1.75	0.74	4.17

*T2, Assessment at discharge; T3, Follow up-assessment 6 months after discharge; HRSD-24, 24-item Version of the Hamilton Rating Depression Scale (20)*.

* *p < 0.05*.

According to the HRSD-24 criterion for treatment response, *z*-test *post-hoc* power analyses revealed that only the following findings yielded acceptable power: the factor “family” on treatment response posttreatment (99%), yet not significant, and the factor “dependence” on treatment response after 6 months of follow-up (87%).

### Secondary Analysis: Prediction of Treatment Response Measured by BDI-II

According to the BDI-II criterion for treatment response, 27 out of the 52 patients (51.9%) met the response criterion at discharge. Six months after discharge, 18 (34.6%) patients still met the response criterion. Table 3 displays the results of the last step of the binary logistic regression using the backward stepwise method for treatment response regarding BDI-II. The factor “dependence” [*b* = −0.81, Wald $\chi_{\left(1\right)}^{2}$ = 3.91, *p* < 0.05] appears to be negatively associated with treatment response at posttreatment, while the Exp(*B*) value indicates that when “dependence” is increased by one unit, the odds ratio is 0.51 times as large and the patients are 49% less likely to respond to treatment. In the 6-month follow-up assessment, the factor “friends” appears negatively related to treatment response [*b* = −0.69, Wald $\chi_{\left(1\right)}^{2}$ = 4.87, *p* = 0.03]. Other factors were not significantly associated with treatment response (*p* > 0.05).

**Table 3:** Results of the logistic regression of negative effects on treatment response at discharge (T2) and follow-up 6 months after discharge (T3) measured by Beck Depression Inventory (BDI-II).

**Variables**	***B***	**Wald**	***p***	**Exp(*B*)**	**95.0% CI for Exp(** ***B*** **)**	
					**Lower**	**Upper**
**Criterion: Response on** ***BDI-II*** **T2**						
Dependence	−0.81	3.91	0.05^*^	0.45	0.20	0.99
**Criterion: Response on** ***BDI-II*** **T3**						
Friends	−0.68	4.87	0.03^*^	0.51	0.28	0.93
Malpractice	0.62	2.28	0.13	1.86	0.83	4.15

*T2, Assessment at discharge; T3, Follow-up-assessment 6 months after discharge; BDI-II, Beck Depression Inventory-II (21)*.

* *p < 0.05*.

According to the BDI-II criterion for treatment response, the power of these models for both posttreatment and after 6 months of follow-up was overall rather low with the factor “dependence” achieving the highest power (74%), yet slightly under the threshold of acceptable power (i.e., 80%).

## Discussion

To date, there are only a few published studies investigating specific negative effects and their impact on the outcome of different specific psychotherapies [exception, e.g., (7)]. A better understanding of the rates of occurrence and relevance of negative effects is also relevant to adequately inform the patient about possible risks of the treatment. This study therefore aimed at (1) assessing the negative effects of a multimodal inpatient CBASP treatment program, as measured by the established and validated instrument INEP (3), and (2) evaluating the impact on treatment response to assess the relevance of negative effects. To achieve the first objective, the reported rates of occurrence of negative effects of the multimodal inpatient CBASP treatment program were examined. Notably, over 90% of patients reported retrospectively to have experienced at least one negative effect during treatment. This finding is consistent with previous studies investigating negative effects in patient populations being treated in outpatient settings (3), but exceeds reported rates of occurrences in inpatient routine clinical care (5, 6). This percentage is also higher than in a recent study that also focused on depressive patients who, however, filled out a different questionnaire than INEP via the Internet and previously underwent outpatient psychotherapy (42). Our comparatively high percentage may be explained by the specific characteristics of patients with treatment-resistant CD, that is, severe symptoms, early onset (age < 21 years), suicidality, and high percentage of reported childhood maltreatment (10, 43, 44), as well as the high-dosage short-term inpatient CBASP program with a strong focus on negative relationship experiences during childhood and the therapist–patient relationship (45). Most frequently in this study, patients reported having developed a dependence on their therapist (almost half of the patients). Stigmatization, financial concerns, and intrapersonal changes due to transient symptom deterioration were reported by one-third of all patients (second most frequent). At first glance, the result that 6.1% of patients reported malpractice appears alarmingly high. A precise analysis of the items that form this scale, however, shows that this comparatively high percentage is due to two items stating that patients felt forced by the therapist to do things they did not want to do, and that patients felt hurt by therapists' statements. In the case of the first item, the patients probably thought mainly of the interpersonal role-plays, which are intended in the CBASP strategy situational analysis in group and individual therapies. Chronically depressed patients usually have difficulties performing the role plays at the beginning of treatment due to their pronounced interpersonal problems (46). In addition, some patients may initially find therapists' statements painful, which are being made in the context of disciplined personal involvement. Therapists address their patients' interpersonally difficult behavior and explain the possible interpersonal consequences, which may initially seem confrontational. The goal, however, is to facilitate long-term healing experiences in relationships. Accordingly, in the course of treatment, patients usually notice how helpful these interpersonal strategies are, which is supported by studies that show that after CBASP therapies, the interpersonal problems have actually decreased (47, 48). Since we could not find any negative correlation to the treatment outcome, such specific malpractice aspects appear to be benign. It should be stressed that 0% reported sexual abuse, physical assaults, or other misconduct. Of note, the subscale malpractice of the INEP showed questionable psychometric properties, for example, only satisfactory internal consistency (3). Concerning the second objective, the results of the regression analyses suggest that, in particular, dependence on the therapist, as the most frequent dimension of negative effects, seems to play a significant role for treatment response on a self- and clinician-rated instrument. While dependence on the therapist is negatively associated with self-rated treatment response defined by BDI-II at discharge, the same factor is negatively linked to clinician-rated treatment response by HRSD-24 also in the long run. Of note, the rate of occurrence of this negative effect dimension is in our study only slightly higher compared to a psychiatric inpatient sample with various mental disorders (5).

In general, adverse event methods seem to be heterogeneous and insufficiently reported in RCTs in CD (49). However, a recent study found that patients receiving supportive psychotherapy reported less severe adverse events in general and less severe adverse events related to personal life and to occupational life than patients receiving CBASP, while less adverse events related to suicidal thoughts were reported in CBASP compared with supportive psychotherapy (50). The authors discussed that the differences in the profile of adverse events may be explained by specific treatment elements, as adverse events related to personal and professional life, for example, may be considered a necessary and expected but temporary adverse treatment outcome of effective CBASP treatment. This is in line with our findings, which underline that most of the reported negative effects had no impact on the treatment outcome. However, given the limitations of this study (see below), our results cautiously suggest that the more a patient reports dependence on her/his therapist, the less likely she/he might benefit from treatment. Yet, there are many possible explanations for this preliminary finding:

- It appears plausible that the high number of personality disorders (61.4% overall, of which 5.7% were diagnosed with a dependent personality disorder) and personality disorder traits (67.1% overall, of which 32.9% were diagnosed with dependent personality disorder traits) of our sample may explain the relatively high percentage of patients reporting dependence on their therapist. Since studies show that personality disorders *per se* are a negative predictor of the outcome of psychotherapy in depressed patients [e.g., (51)], they might function as the underlying factor being responsible for the finding that the reported dependence on the therapist is negatively related to treatment outcome.
- Notably, it could also be argued that the dependence factor is not a side effect, but simply a consequence of a poor therapeutic alliance during treatment. Since psychotherapy research has often confirmed that a positive therapeutic alliance is associated with a positive outcome [e.g., (15)], dependence as an indicator of a negative alliance could explain the worse response. However, it has recently been reported that patients' dependency on mental healthcare seems to be associated with a better therapeutic alliance (52). Indeed, a relationship between the therapeutic alliance and outcome in CBASP has been well-established in research (17, 18, 21), while in particular a positive early therapeutic alliance predicted beneficial outcomes in CBASP (19, 20). However, a history of drug abuse/dependence and lower past and lower current social adjustment predicted a significantly poorer therapeutic alliance in CBASP (53).
- In addition, the level of severity of the personality dimension “dependency” may have a differential influence on the treatment outcome. Interestingly, a recent study investigated the impact on treatment outcome of the personality dimension dependency according to Blatt (54) in treatment-resistant chronically depressed patients and found that patients with more maladaptive dependent features did not benefit from a long-term psychoanalytic psychotherapy (LTPP) or treatment as usual (TAU), while those with less maladaptive dependent features showed considerable gains from LTPP but not from TAU (55).
- The specific strategies of CBASP might trigger dependence on the therapist. Notably, many patients suffering from CD reported to have experienced both childhood maltreatment [e.g., (10, 44)] and current interpersonal problems such as submissive or hostile behavior (46, 47) or emotional and behavioral avoidance (56, 57). The association between childhood maltreatment and interpersonal problems has recently been reported (58). CBASP-specific techniques (in particular the disciplined personal involvement and the interpersonal discrimination exercise) may allow those patients to experience new healing and corrective relationships—sometimes for the first time in their lives, characterized by predictability, interpersonal closeness, and warmth. Conversely, this new experience could also initially promote dependence on their therapists, especially when isolated patients have no other positive significant others in their life. Of note, CBASP traditionally highlights the importance of using autonomy-promoting strategies such as to encourage patients to write out a complete sentence in the situational analysis and to stress the use of the patient's own wording in an intervention. While autonomy has been well-promoted within the therapeutic relationship, interpersonal change and avoidance behavior outside of treatment (e.g., in occupational and private life) were possibly not yet sufficiently addressed because of the limits of the specific inpatient treatment setting and a lack of transfer opportunities.
- The individual psychotherapy in this CBASP treatment program was delivered in a high intensity with two CBASP 50-min sessions per week that probably have fostered dependence. Additionally, the high intensity of social encounters between the entire team and the patients on the ward (e.g., group psychotherapy twice a week, nurse–patient encounters, and social worker contact) for a predominantly socially isolated patient group of chronic depressive patients with interpersonal dysfunctions might have contributed to an increased dependence, since the main phase of this CBASP treatment focused on the use of the Kiesler circle (e.g., enhancing the understanding of their stimulus character and impact on others) and on conducting situational analyses with subsequent role-playing events to modify inappropriate behavior using the potential of other patients in the group psychotherapy.
- Finally, the applied intensive multimodal inpatient CBASP treatment program was limited to 12 weeks. Patients who have experienced dependence on their therapist may not feel sufficiently prepared yet for the demands of daily life at the end of this comparatively short treatment period, which may lead to an unfavorable treatment outcome. However, the finding of an increased dependence might have been at least partially confounded by the individual aftercare plan as four-fifths of the participants received outpatient psychotherapy, yet one-third of those underwent CBASP, after discharge.

Moreover, our results showed that negative effects related to intrapersonal changes appear negatively related to treatment response defined by HRSD-24 at discharge. This result cautiously indicates that the more a patient has suffered from intrapersonal changes (like transient deterioration of symptoms) during therapy, the less likely he/she might benefit from the therapy in the short term, but not in the long term. Lastly, the result that negative effects on friends are negatively related to long-term treatment response defined by BDI-II after 6 months may indicate that the more a patient reports negative effects on friends caused by treatment, the less he/she may improve in terms of treatment response. This result may be interpreted against the background of theories and approaches that consider CD primarily as a relationship disorder (8).

Although taking into account that two-tailed analyses yield lower power in general, the results of the *post-hoc* power analyses however indicated that solely the finding of the factor dependence on treatment response seems to be relatively robust and should therefore be interpreted.

### Limitations and Future Research

The interpretability of the results of this study is reduced by some limitations. First, the INEP data were collected exclusively retrospectively during a follow-up period and the period between discharge and INEP survey varied between 6 and 12 months. Thus, recall effects such as memory bias and the primacy–recency effect, forgetfulness, retrieval errors, or important experiences occurring after treatment had been completed may have distorted the reported negative effects. Indeed, subjectively experienced negative effects of (group) psychotherapy seemed to decrease in the course of treatment (6). For example, a bias toward the course of the depression after the end of treatment is conceivable, whereby a positive symptom course after treatment could lead to a more positive assessment of the received treatment with fewer negative effects. On the other hand, negative effects of inpatient treatment programs could actually only occur after discharge, whereby these in particular could possibly be detrimental and therefore valuable to record. Future studies should distinguish between the assessment of negative effects during and after treatment to have a more nuanced profile of negative effects in the short and long run. Furthermore, the hindsight bias must be considered when interpreting the results of this study, that is, that patients who did not respond see their treatment in a less positive light and report more negative effects. This retrospective evaluation of negative effects also means that the short-term outcome was recorded before the evaluation of the negative effects. Therefore, the analyses should also be interpreted with caution, as the chronological sequence of the recording of statistical predictors before the variable to be predicted (here: outcome) could not be fulfilled in this way. It is essential that future studies should record negative effects regularly in the therapy process and at uniform measurement times. Future large-scale studies should integrate the assessment of negative effects of psychological interventions in the data collection and analysis design when planning the study as proposed by new guidelines (59). Secondly, INEP does not include any specific negative effects of an inpatient setting, such as group therapy sessions or conflicts with other patients or staff, nor does it consider the influence of pharmacotherapy, which should be directly addressed in future research. Moreover, INEP does not simultaneously capture positive effects as the Positive and negative Effects of Psychotherapy Scale (PANEPS) instrument does (7, 42), which is why this study could not examine the relationship between negative and positive effects (apart from the outcome). Future studies should therefore use a measurement that captures both positive and negative effects [e.g., by using the PANEPS; (40)] to further minimize priming and associated potential nocebo effects. However, one positive aspect of the INEP is the bipolar response format, which records not only deteriorations but also improvements or missing changes, thus partly preventing negative priming (3). Thirdly, although there are promising findings underlining the seven-factor structure of INEP (3), these seven factors still lack some psychometric evaluations. Fourthly, future studies should exclusively use the DSM-5 criteria and the term persistent depressive disorder. Yet, since this study was still conducted under the term of chronic depression, this term was used throughout our manuscript and in reference to the main outcome paper (14). For a diagnostic cross-walk, we refer to relevant literature [e.g., (60, 61)]. Finally, the lack of a control group, additional algorithm-based pharmacotherapy, and a relatively small sample size generally complicate the ability to interpret the results. Due to the lack of a control group, we could not rule out that negative effects could also be due to psychotherapy *per se*, and not specifically due to the inpatient CBASP treatment. As we investigated a multimodal inpatient CBASP program including multiple interventions and therapists, it is difficult to determine the percentage of variance attributable to individual CBASP sessions. However, since all members of the treatment team were trained in CBASP, the CBASP-specific techniques could also be used by all therapists in their respective therapies (e.g., disciplined personal involvement with interpersonal discrimination exercises). Compared to many inpatient psychotherapy programs, the intensity of CBASP can therefore be classified as very high, as patients also received 2 individual sessions per week over the course of 12 weeks leading to a total number of 24 sessions on average. However, this high CBASP intensity could have contributed to the findings.

## Conclusions

In this study, a multimodal inpatient CBASP treatment program seems to be associated with negative effects, which may be explained by the specific characteristics of patients with treatment-resistant CD and the focus of CBASP techniques on the patient–therapist relationship. Interestingly, most reported negative effects do not appear to have an impact on treatment outcome. However, dependence on the therapist, as the most frequent dimension of negative effects, seems to be negatively linked to both observer- and self-rated treatment response. If large randomized controlled trials find that CBASP is more likely to trigger dependence on the therapist than other psychotherapy concepts and that this perceived dependence actually has a negative impact on outcomes, then clinical implications such as prolonging treatment and focusing more on self-help and autonomy of the patient should be considered.

## Data Availability Statement

The raw data supporting the conclusions of this article will be made available by the authors, without undue reservation.

## Ethics Statement

The studies involving human participants were reviewed and approved by Ethics Committee of the University of Freiburg. The patients/participants provided their written informed consent to participate in this study.

## Author Contributions

PH originally drafted and edited the manuscript. E-LB supervised, reviewed, and edited the manuscript. SH was significantly involved in the computation of the statistical analyses and visualization of the results. CN was responsible for the acquisition of these data for the present work. All authors contributed to the article and approved the submitted version.

## Conflict of Interest

The authors declare that the research was conducted in the absence of any commercial or financial relationships that could be construed as a potential conflict of interest.

## Publisher's Note

All claims expressed in this article are solely those of the authors and do not necessarily represent those of their affiliated organizations, or those of the publisher, the editors and the reviewers. Any product that may be evaluated in this article, or claim that may be made by its manufacturer, is not guaranteed or endorsed by the publisher.

## Footnotes

^1^The domain “intrapersonal changes” describes in the broadest sense negative effects on emotional experience and social functioning (3).
